# A Path to Qualification of PET/MRI Scanners for Multicenter Brain Imaging Studies: Evaluation of MRI-Based Attenuation Correction Methods Using a Patient Phantom

**DOI:** 10.2967/jnumed.120.261881

**Published:** 2022-04

**Authors:** Ciprian Catana, Richard Laforest, Hongyu An, Fernando Boada, Tuoyu Cao, David Faul, Bjoern Jakoby, Floris P. Jansen, Bradley J. Kemp, Paul E. Kinahan, Peder Larson, Michael A. Levine, Piotr Maniawski, Osama Mawlawi, Jonathan E. McConathy, Alan B. McMillan, Julie C. Price, Abhejit Rajagopal, John Sunderland, Patrick Veit-Haibach, Kristen A. Wangerin, Chunwei Ying, Thomas A. Hope

**Affiliations:** 1 Athinoula A. Martinos Center for Biomedical Imaging, Department of Radiology, Massachusetts General Hospital and Harvard Medical School, Charlestown, Massachusetts;; 2 Mallinckrodt Institute of Radiology, Washington University School of Medicine, St Louis, Missouri;; 3 Center for Advanced Imaging Innovation and Research, Department of Radiology, New York University Langone Medical Center, New York, New York;; 4 Shanghai United Imaging Healthcare Co., Ltd., Shanghai, China;; 5 Siemens Medical Solutions USA, Inc., Malvern, Pennsylvania;; 6 Siemens MR, Siemens Healthcare GmbH, Erlangen, Germany;; 7 PET/MR Engineering, GE Healthcare, Chicago, Illinois;; 8 Division of Nuclear Medicine, Mayo Clinic, Rochester, Minnesota;; 9 Imaging Research Laboratory, University of Washington, Seattle, Washington;; 10 Department of Radiology and Biomedical Imaging, University of California, San Francisco, California;; 11 Advanced Molecular Imaging, Philips Healthcare, Cleveland, Ohio;; 12 Department of Imaging Physics, University of Texas M.D. Anderson Cancer Center, Houston, Texas;; 13 Department of Radiology, University of Alabama at Birmingham, Birmingham, Alabama;; 14 Department of Radiology, University of Wisconsin School of Medicine and Public Health, Madison, Wisconsin;; 15 Division of Nuclear Medicine, Department of Radiology, University of Iowa, Iowa City, Iowa;; 16 Toronto Joint Department of Medical Imaging, University Health Network, Sinai Health System, and Women’s College Hospital, Department of Medical Imaging, University of Toronto, Toronto, Canada;; 17 GE Healthcare, Chicago, Illinois; and; 18 Department of Biomedical Engineering, Washington University in St. Louis, St. Louis, Missouri

**Keywords:** PET/MRI, attenuation correction, multicenter trials, qualification

## Abstract

PET/MRI scanners cannot be qualified in the manner adopted for hybrid PET/CT devices. The main hurdle with qualification in PET/MRI is that attenuation correction (AC) cannot be adequately measured in conventional PET phantoms because of the difficulty in converting the MR images of the physical structures (e.g., plastic) into electron density maps. Over the last decade, a plethora of novel MRI-based algorithms has been developed to more accurately derive the attenuation properties of the human head, including the skull. Although promising, none of these techniques has yet emerged as an optimal and universally adopted strategy for AC in PET/MRI. In this work, we propose a path for PET/MRI qualification for multicenter brain imaging studies. Specifically, our solution is to separate the head AC from the other factors that affect PET data quantification and use a patient as a phantom to assess the former. The emission data collected on the integrated PET/MRI scanner to be qualified should be reconstructed using both MRI- and CT-based AC methods, and whole-brain qualitative and quantitative (both voxelwise and regional) analyses should be performed. The MRI-based approach will be considered satisfactory if the PET quantification bias is within the acceptance criteria specified here. We have implemented this approach successfully across 2 PET/MRI scanner manufacturers at 2 sites.

## Visual Abstract

**Figure:**
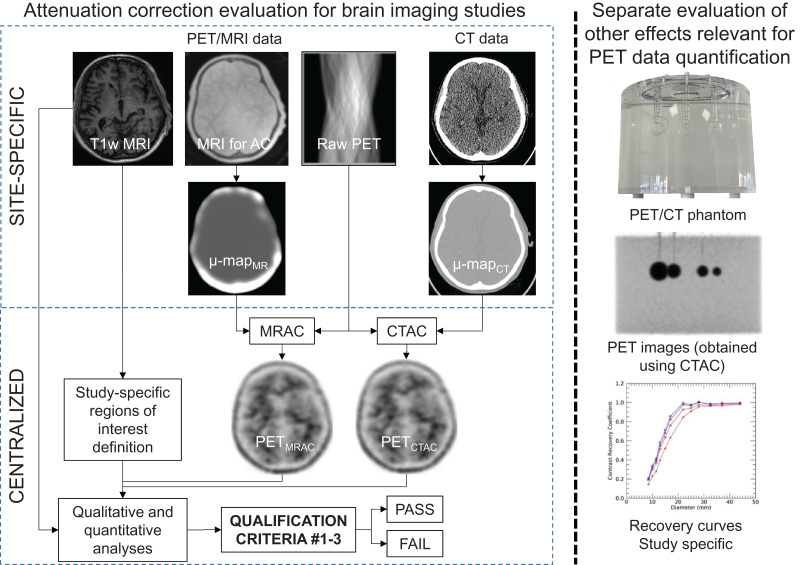

Simultaneous PET/MRI scanners were introduced commercially for human imaging in 2010 and have since made their way into research laboratories and clinics following in the footsteps of hybrid PET/CT, which saw its introduction in early 2000. In contrast to PET/CT, which experienced rapid clinical acceptance by adding much needed high-resolution anatomic information and faster attenuation correction (AC) to functional and molecular imaging, combined PET/MRI has seen a much slower acceptance. In addition to the higher cost of the modality, one of the reasons for this slower adoption has been the fact that AC is more challenging (*1*) because bone tissue cannot easily be imaged by MRI and may be misclassified, resulting in quantitative uncertainties that have helped to perpetuate the viewpoint that PET/MRI remains investigational. Over the last decade, several MRI-based algorithms have been developed to more accurately derive the PET (511 keV) attenuation properties of the human head, including the bone tissue. Algorithms such as ultrashort time of echo, zero time of echo (ZTE; GE Healthcare), atlas-based, or, most recently, machine learning approaches have been proposed to replace or complement the vendor-provided 2-point Dixon (or LAVA Flex; GE Healthcare) sequence that is routinely used in clinical settings (*1*,*2*). These MRI-based AC (MRAC) algorithms have been evaluated by imaging patients sequentially on PET/CT and PET/MRI scanners and using the CT-based AC as the gold standard. Considering these developments and the need for scanner validation, a clear path to the qualification of this modality is both timely and necessary. Although methods to perform a transmission scan inside the PET/MRI scanner have also been proposed (*3*–*5*) and could also be used for validating MRAC approaches, they require additional hardware and expertise.

A related limitation to clinical acceptance and inclusion in clinical trials has been that PET/MRI scanners cannot be qualified in the manner adopted for PET/CT. PET/CT qualification or scanner validation, for purposes ranging from clinical use to participation in a clinical trial with PET quantitative endpoints, typically proceeds with scanning of standardized phantoms (filled with a radiotracer mixed with water) of predefined geometry such as the American College of Radiology (ACR), Clinical Trials Network, or National Electrical Manufacturers Association International Electrotechnical Commission phantoms. For PET/CT scanners, this works well because the linear attenuation coefficient of water is close to that of soft tissue for both PET and CT. Additionally, CT provides sufficient information to infer the linear attenuation coefficients of other materials (*6*). These traditional phantoms, however, cannot be imaged accurately by MRI because proton properties in magnetic fields do not readily translate to electron density, atomic structure, and 511-keV photon attenuation. Specifically, the transverse relaxation time (T2) of protons in phantom materials such as plastic is too short to be captured conventionally, leading to little measurable signal from nearly all types of MRI pulse sequences. Although substantial progress has been made in manufacturing phantoms capable of mimicking both electron density and MRI contrast characteristics of human tissues (*7*–*9*), no such phantom that could be used to assess the performance of multiple MRAC techniques is yet widely available. Additionally, water-filled phantoms, a mainstay in the accreditation of PET scanners (*10*), produce resonance artifacts in MR images (*11*). In other words, although the standard phantoms accurately replicate the imaging physics of PET and CT for patients, the same is not true for MRI.

In this work, we propose a path for PET/MRI qualification for brain imaging studies using a patient as a phantom. We explain the differences between accreditation and qualification, outline the need for both, review the accreditation and qualification process in the context of PET/CT, and describe the proposed solution in terms of data acquisition and analysis and the definition of qualification criteria.

## ACCREDITATION AND QUALIFICATION

The term *accreditation* is used primarily in the clinical setting. For example, all centers in the United States that bill for nuclear medicine procedures are required to be accredited to receive all the reimbursement from Medicare. The term *qualification* describes the process of determining whether a specific scanner can be used in the setting of a specific clinical trial. Frequently, contract research organizations will require specific phantom imaging tests to qualify scanners before allowing sites to enroll in imaging trials. In many settings, approaches to accreditation are used as part of a qualification process. There are many organizations that provide qualification services in the setting of multicenter clinical trials (*12*,*13*). Though the terms *accreditation* and *qualification* are often used interchangeably, it is important to understand the distinctions between them. The goal of this article is to propose an approach to PET/MRI qualification for brain studies, such that these devices can be used for multicenter clinical trials.

## APPROACHES USED IN PET/CT

One of the most commonly used means of accreditation in PET/CT is the ACR accreditation program (*10*,*14*). The ACR accreditation program defines the requirements for the personnel performing and interpreting the study, quality control, and peer review. Additionally, each site must provide images of a specific PET phantom and clinical images that are reviewed centrally. The phantom and clinical images are evaluated qualitatively before accreditation. The phantom images have specific quantitative acceptance criteria. For example, ACR requires ±15% error in the SUV of the background (as well as other requirements for contrast recovery). Accreditation does not define the performance of the procedure (e.g., uptake time and injected activity) but rather focuses on the facility, personnel, device, and resultant images. Other organizations also provide accreditation services, such as the Intersocietal Accreditation Commission, RadSite, and the Joint Commission (*15*,*16*).

For qualification, many clinical trials will accept ACR accreditation, but those focused on novel radiotracers or quantitative PET measures frequently require more stringent approaches, which can overlap with harmonization. *Harmonization* is a term that describes setting up the image acquisition and reconstruction parameters so that approximately the same quantitative outcomes are obtained independently of scanners; this approach is sometimes used in trials with quantitative primary or secondary endpoints. Two main approaches for harmonization are those put forth by the Clinical Trials Network and the European Association of Nuclear Medicine Research Ltd. (*13*,*17*). These approaches use phantoms with spheres of varying sizes, filled following exact phantom preparation procedures, to determine harmonized reconstruction parameters capable of producing quantitative results that yield measured SUVs within a predetermined range. Using these approaches, one can minimize variability in PET quantification across imaging devices.

All accreditation, qualification, and harmonization procedures in PET/CT require the imaging of a phantom filled with a known quantity of radiotracer in a water solution.

## PROPOSED SOLUTION FOR PET/MRI SCANNERS: QUALIFICATION USING HUMAN PHANTOMS

Given the above-mentioned challenges in imaging standard phantoms, a more manageable approach to PET/MRI qualification is to evaluate the PET reconstruction pipeline’s constituent parts independently. Specifically, the challenge in the generation of the attenuation map can be isolated from the other effects that influence the PET quantification (i.e., such corrections as those for randoms, dead time, and decay and those related to the image reconstruction). To address the former challenge, we propose to use patients scanned sequentially on both CT or PET/CT and PET/MRI as phantoms and evaluate the difference in the resultant attenuation maps and impact on PET data quantification. This approach builds on the methodology typically used for validating MRAC using CT-based AC as the standard. We propose the below procedures to standardize this approach so it can be used to qualify a particular PET/MRI scanner. The guideline recommended here is specific to the head but could in principle be adapted to other more complex regions, although additional challenges would obviously need to be considered for whole-body applications (*1*). Other factors relevant for the qualification of the PET component of the integrated PET/MRI scanner in a multicenter trial will be assessed using separate imaging phantoms and procedures already in place for PET/CT.

### Data Acquisition

#### CT Data Acquisition

A noncontrast CT study should be performed using parameters typically used for AC in PET scans or diagnostic examinations according to the clinical protocols, and the images should be reconstructed using standard algorithms (e.g., analytic filtered backprojection and iterative techniques). The subject should be positioned on the CT scanner with the arms outside the field of view (i.e., arms down, as is typically done for head PET/CT and PET/MRI examinations), and the entire head should be scanned (i.e., from the top of the head to the lower neck). Patients with metallic implants should not be used as they could bias both the CT-based and the MRI-based attenuation maps. Additionally, subjects should be excluded if significant artifacts (e.g., streaks, motion, or scanner malfunction) are seen in the CT images.

#### MRI Data Acquisition

MRI data should be acquired using the radiofrequency coil that will be used in the clinical study or clinical trial. The site-specific MRI sequence used for generating the attenuation map (e.g., Dixon–volumetric interpolated breath-hold examination, magnetization-prepared rapid acquisition with gradient echo, ZTE, or ultrashort time of echo) should also be acquired with the same parameters as those used in the clinical trial. The whole head (including nose and ears) and the part of the neck present in the physical PET field of view should be covered. If the site-specific MRAC method is different from the vendor-specific one, the vendor-specific MRAC sequences should also be acquired. Additionally, a vendor-specific sequence for obtaining high-resolution morphologic MRI data (e.g., magnetization-prepared rapid acquisition with gradient echo or BRAVO [GE Healthcare] sequences with approximately 1 mm^3^ resolution and maximum 1.5-mm slice thickness) should be acquired for the purposes of image registration to the CT scan and region-of-interest (ROI) definition. Any MR images with artifacts that are known to bias the PET data quantification (e.g., susceptibility, water–fat inversion, ghosting, or motion) should be excluded from the evaluation. Dental fillings, which might be present in many subjects, do not usually lead to significant artifacts and would not be excluded. The MRI-based attenuation map should be generated using the site-specific algorithm to be used in the clinical trial (either developed in-house or provided by the manufacturer).

#### PET Data Acquisition

The radiotracer used for evaluation will depend on the specific study. The emission data should be acquired using the integrated PET/MRI in one of the following 2 ways: PET/CT followed by same-day PET/MRI or CT-only followed by same-week PET/MRI. In the first scenario, the subject should undergo the additional PET/MRI examination within a reasonable time specific to the radiotracer to provide adequate counts in the PET data acquired on the PET/MRI device (e.g., within 3 h from the time of ^18^F-FDG administration). The emission data acquired as part of the PET/CT examination are not used in the analysis, as the focus is on analyzing the impact on the PET data quantification acquired on the PET/MRI scanner. In the second scenario, the PET/MRI examination should be scheduled within 1 wk of the clinical CT scan. As significant changes could occur within a week even without surgical interventions (e.g., differences in the filling of the sinuses could introduce bias in adjacent gray matter structures), subjects with a recent onset of upper respiratory infections, acute sinusitis, and other such conditions should be excluded. The acquisition duration on the PET/MRI device should be at least 10 min in both scenarios, and the emission data should be saved in a manner that permits retrospective reconstruction (i.e., list mode or sinograms, plus associated data for corrections).

In both cases, the patients should be scanned with arms down and the head positioned in the MRI scanner as similarly as possible to the CT scan. Specifically, the technologist should review the CT images and try to position the head in a similar orientation with respect to the neck (e.g., no head lateral rotation and a similar degree of flexion) to minimize the need for nonrigid body registration. Additionally, the head should be centered in the PET axial field of view to ensure full coverage in a single acquisition.

### Centralized Data Processing and Analysis

We recommend the creation of a PET/MRI scanner accreditation group or organization to perform the steps described below. This group should have the capability to process and analyze the data collected on any of the PET/MRI scanners and reproduce all the steps described below using the following data: site-specific MRI-based attenuation maps, morphologic MR images, CT images (or CT-based attenuation map), and raw emission data in sinogram or list-mode format and the additional files required for image reconstruction (e.g., normalization file and hardware attenuation maps).

To minimize the contribution of factors not related to the MRI-based attenuation map generation procedure, the accreditation group will use software provided by the manufacturers or freely available packages to standardize the following steps.

### Data Processing

#### CT Data Processing

First, the patient bed and head holder will be removed from the CT images using vendor-provided software. Second, the CT volume will be coregistered to the morphologic high-resolution MRI volume using rigid body registration with normalized mutual information as the objective function (e.g., using Elastix (*18*,*19*), Statistical Parametric Mapping (*20*), Insight Toolkit (*21*,*22*), or similar software). The accuracy of the coregistration will be assessed visually by an experienced reader. Third, the Hounsfield units will be converted to linear attenuation coefficients at 511 keV using the vendor-specific procedure. Fourth, the resulting CT-based attenuation maps will be smoothed using a gaussian filter (with a kernel size that ensures the resulting attenuation maps match the PET scanner spatial resolution) and resampled into the PET space of the specific PET/MRI device. If the CT-based attenuation map is incomplete (because of the shorter axial coverage in the neck region or different positioning between the 2 examinations), the missing data will be copied from the MRI-based attenuation map. Finally, the attenuation map will be exported in a format that allows its use for AC using the standard PET image reconstruction pipeline.

#### PET Data Processing

The PET images will be reconstructed with the reconstruction algorithm used in the clinical trial, applying both the CT-based AC and the MRAC maps created above. Typically, the scatter correction provided by the manufacturer will be used in both cases (although the attenuation map is usually used for scatter estimation, only the joint impact of both attenuation and scatter corrections on PET data quantification is of interest here). Postreconstruction smoothing will be applied according to the study protocol.

### Data Analysis

#### ROI Definition

Subject-specific ROIs will be defined from the morphologic MRI data using FreeSurfer (*23*). A representative subset of study-specific ROIs will be selected for regional data analysis. Additionally, a brain mask (i.e., all the voxels corresponding to gray and white matter) will be obtained from the MRI data.

#### Quantitative Evaluation of the Attenuation-Corrected PET Data

The bias present in the PET images reconstructed with MRAC relative to those reconstructed with CT-based AC will be assessed by computing the voxelwise percentage differences throughout the whole brain mask (i.e., all the voxels corresponding to brain tissue). Additionally, a regional analysis will be performed using the FreeSurfer-defined ROIs. Average percentage differences, as well as average absolute percentage differences, will be computed for all selected ROIs.

### Qualification Criteria (QC)

#### QC 1

The MRI-based attenuation maps and corresponding PET images should be free of artifacts (e.g., fat–water inversion, susceptibility artifacts in the MRI-based map or streak artifacts in the CT-based map, and incomplete head coverage), and no obvious misregistration should be noted in the overlaid images.

#### QC 2

The voxelwise relative differences between the PET images attenuation corrected using the MRAC and CT-based approaches should be below 10% in at least 90% of the voxels included in the brain mask.

#### QC 3

The average absolute percentage differences between the PET images attenuation corrected using the MRAC and CT-based approaches should be below 10% in all study-specific ROIs.

#### QC 4

For studies involving reference tissue analysis (e.g., SUV ratios for amyloid PET imaging in neurodegeneration), a more stringent threshold could be set for the reference ROI (e.g., less than 5% bias in the cerebellum in the case of amyloid PET imaging).

## EXAMPLE APPLICATION

### Methods

The procedures described above were followed for acquiring, processing, and analyzing the data to qualify 2 different PET/MRI scanners for a hypothetical study aimed at assessing β-amyloid accumulation in Alzheimer disease subjects. A total of 10 datasets were assessed, obtained from 5 subjects scanned on the Biograph mMR and 5 on the Signa PET/MRI scanners. The results from representative cases are discussed below.

At 1 institution (UCSF), subjects underwent ^18^F-AV-45 (florbetapir) imaging using the Signa PET/MRI and Discovery STE PET/CT scanners (GE Healthcare). The MRI-based attenuation maps were generated using atlas- (*24*,*25*) and ZTE-based (*26*,*27*) approaches. At the other institution (Washington University in St. Louis), subjects underwent ^18^F-AV-45 imaging using the Biograph mMR and Biograph Vision PET/CT scanners (Siemens Healthineers). The MRI-based attenuation maps were generated using the Dixon- (*28*) and skull model-based (*29*) approaches. The FreeSurfer-derived cortical ROIs were combined into 4 study-specific large bilateral regions (frontal, cingulate, parietal, and lateral temporal) previously proposed for assessing β-amyloid deposition in this patient population (*30*,*31*). Additionally, bilateral regions corresponding to white matter and whole cerebellum were defined.

### Results

**FIGURE 1.**
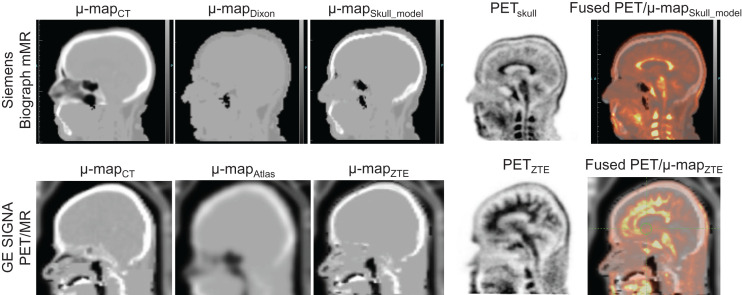
Attenuation maps and corresponding ^18^F-AV-45 PET images for 2 representative subjects. No artifacts or obvious misregistration can be observed (QC 1).

The attenuation maps and the corresponding PET images for 2 representative subjects free of artifacts and properly registered are presented in Figure 1 and Supplemental Figures 1 and 2 (supplemental materials are available at http://jnm.snmjournals.org) (QC 1).

**FIGURE 2.**
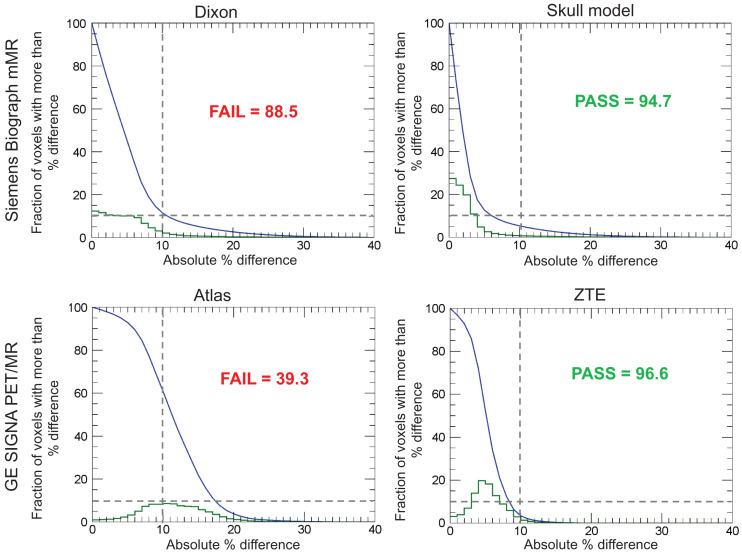
Cumulative voxelwise relative differences between PET images obtained using 4 attenuation map generation methods and those generated using reference CT-based approach for 2 representative subjects (blue). Voxelwise relative differences are below 10% in at least 90% of voxels included in brain masks for Siemens skull model-based and GE ZTE-based approaches (QC 2). Percentage of voxels with absolute relative difference smaller than 10% is indicated in each case. Green line represents histogram of pixelwise percentage difference.

Figure 2 shows the cumulative pixelwise absolute difference histogram (blue) and pixelwise absolute percentage difference histogram (green) for Dixon- and skull model-based AC on the Biograph mMR, and atlas- and ZTE-based AC on the Signa PET/MRI scanners. The relative differences between the PET images obtained using the Siemens skull model- and ZTE-based methods with respect to the CT-based approach were below 10% in 94.67% and 96.59% of the voxels included in the brain mask, respectively (QC 2). On the other hand, the Siemens Dixon- and GE atlas-based approaches did not meet this acceptance criterion.

**FIGURE 3.**
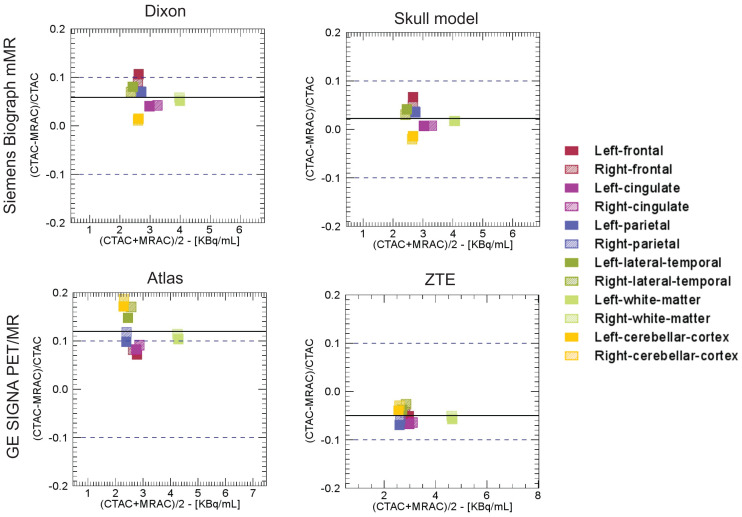
Bland–Altman plots of absolute relative differences between PET images obtained using 4 attenuation map generation methods and those generated using reference CT-based approach for study-specific FreeSurfer-based ROIs for 2 representative subjects. Average absolute percentage differences are below 10% in all study-specific ROIs for Siemens skull model-based and GE ZTE-based approaches (QC 3). CTAC = CT-based AC.

The regional absolute relative differences were below 10% for all the study-specific ROIs described above for the Siemens skull model- and ZTE-based approaches as depicted in the Bland–Altman plots shown in Figure 3 (QC 3). The Siemens Dixon- and GE atlas-based approaches did not meet this acceptance criterion.

**FIGURE 4.**
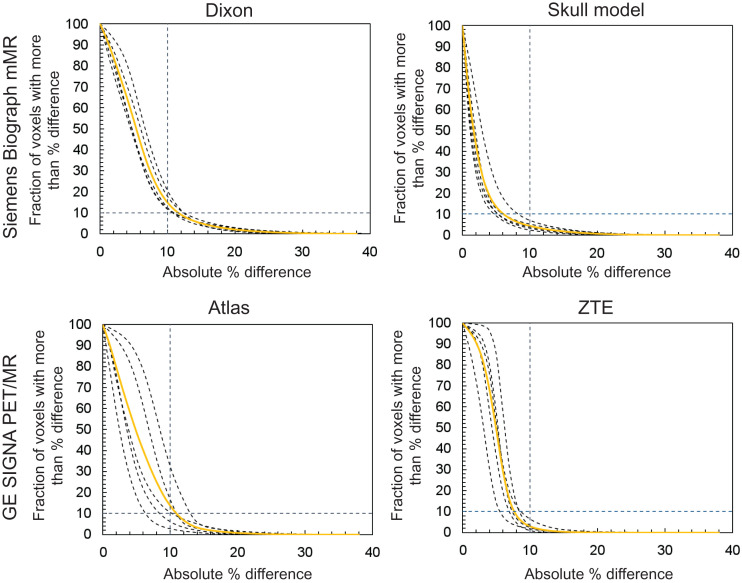
Cumulative voxelwise relative differences between PET images obtained using MRI-based methods with respect to CT-based approach for all 10 subjects analyzed. Solid curve represents mean across 5 subjects for each method. Voxelwise relative differences are below 10% in at least 90% of voxels included in brain masks for all subjects when using Siemens skull model-based and GE ZTE-based approaches.

Plots of cumulative histograms of absolute pixelwise differences and a summary report for all 10 subjects included in the analysis are given in Figure 4 and Supplemental Table 1. The relative differences between the PET images obtained using the Siemens skull model- and ZTE-based methods with respect to the CT-based approach were below 10% in more than 90% of the voxels included in the brain mask for all subjects (QC 2). On the other hand, the Siemens Dixon- and GE atlas-based approaches did not meet this acceptance criterion for 5 and 3 of the subjects, respectively.

## DISCUSSION

We have proposed to use the patient as a phantom to qualify PET/MRI scanners for brain imaging multicenter trials. Because of the absence of suitable phantoms to evaluate MRAC methods, patient phantoms provide the fastest path forward to evaluating quantitative errors associated with AC. The main advantage to this approach is that it will remain robust independently of the MRAC methodology over time. It is important to note that site qualification for evaluating reconstruction methods is still required; this can be done using standard PET/CT phantoms.

Although a study-specific radiotracer is preferred, ^18^F-FDG may also suffice in many indications because of its global uptake pattern, making the assessment of bias from the MRAC generalizable to most other radiotracers relevant to neurologic applications. Although the evaluation methods and qualification criteria were defined to ensure that the assessment is applicable across radiotracers, additional radiotracer- or patient population–specific assessments could be defined and performed if needed. Finally, the proposed methods are also applicable to AC methods that use the emission data to estimate the attenuation map (*32*), as well as the latest generation of machine learning approaches (*33*), including those methods that generate attenuation- and scatter-corrected images directly from the noncorrected images without needing to generate an attenuation map (*34*).

Given the complexity of this method compared with the one applied for qualifying PET/CT scanners using innate phantoms, we have recommended that the data processing be performed by the accreditation group or organization. Although each site would have to submit images and raw data, this task does not require advanced software or training. The centralized processing would ensure that all the steps are performed consistently. The differences between the offline and online data-processing tools could be minimized by obtaining from the equipment manufacturers the tools corresponding to the software version installed on the scanner to be qualified. Furthermore, the PET images attenuation-corrected using the CT- and MRI-based attenuation maps would be reconstructed using the same input parameters. For these reasons, the remaining differences between the offline and online reconstructions would not affect the quantitative evaluation of the AC procedure, which is our only goal here. Other effects relevant for PET data quantification would be assessed using images of standard phantoms (e.g., National Electrical Manufacturers Association or SNMMI Clinical Trials Network phantom) reconstructed using the online tools to ensure that the images meet study-specific criteria such as those related to image uniformity, spatial resolution, and image quality.

One limitation of the proposed approach is that it requires CT data to be acquired either onsite or at a different facility. Furthermore, the need to perform 2 examinations places additional burden on the participants, staff and increases the costs compared with scanning an innate phantom. The radiation exposure is also increased in the CT-only followed by PET/MRI examination scenario.

Another drawback to the proposed approach is that each imaging center is required to transfer raw data (i.e., list-mode or sinogram PET data) to a central processing site to have the dataset reconstructed using both CT- and MRI-based attenuation maps. Sites may not be immediately familiar with how to access and export these large datasets. There is also potential variability associated with CT-to-MRI registration. Nonetheless, in our initial evaluation of the proposed approach we were able to successfully implement the process across 2 centers using 2 different PET/MRI manufacturers, with comparable results. Further work needs to be performed to automate the analysis and to minimize the burden on the central site.

The proposed solution was here applied to the brain, but future work will focus on extending the patient phantom to other parts of the body as accurate MRAC approaches become available. Regional analysis and the impact of MRAC on focal lesion uptake would have to be defined outside the brain (as well as for assessing the impact of AC in the presence of bone lesions in the head). This translation to other body regions will also be facilitated by using a patient phantom, as new phantom geometries do not need to be developed.

Lastly, this approach uses a best-case-scenario patient selected by the individual site, as is done with other qualification approaches, but it does not evaluate the variability across patients. The goal of this qualification approach is to demonstrate that the MRAC methods used on the site-specific scanner are functioning as expected on the basis of manufacturer recommendations. As with all qualification approaches, this approach does not prevent errors in PET quantitation due to large patient-level abnormalities. It was also not our goal to propose a guideline for harmonization of AC methods. However, the proposed method could be adapted for this purpose although that would require different data acquisition and processing protocols (e.g., scanning the same subject on different PET/MRI scanners).

## CONCLUSION

We have proposed a solution for qualifying PET/MRI scanners for brain imaging clinical trials. The most significant challenge is to develop PET/MRI-specific phantoms that are applicable across different MRAC approaches. To address this issue, we have proposed using the patient as a phantom, whereby the scaled CT attenuation map is used to validate the MRI-based map generated for the same patient. The approach was successfully implemented across 2 PET/MRI scanner manufacturers at 2 sites.

## DISCLOSURE

This work was partially supported by the National Institutes of Health (grants 5R01CA212148 and 1U01EB029826). Tuoyu Cao is employed by United Imaging Health Care, David Faul and Bjoern Jakobi are employed by Siemens Healthineers, Floris Jansen and Kristen Wangerin are employed by GE Healthcare, and Piotr Maniawski is employed by Philips Healthcare. No other potential conflict of interest relevant to this article was reported.

## Notes

KEY POINTS **QUESTION:** Can patient phantoms be used to test the head MRAC methods to qualify PET/MRI scanners for multicenter trials? **PERTINENT FINDINGS:** In this consensus paper, we proposed to use a patient as a phantom to assess the accuracy of MRAC using CT as the reference standard. Following the proposed guidelines for data acquisition, image reconstruction, and data analysis, we have tested the proposed approach successfully across 2 PET/MRI scanner manufacturers at 2 sites. **IMPLICATIONS FOR PATIENT CARE:** Integrated PET/MRI scanners can be qualified for multicenter trials focused on neurologic applications.
